# The cellular distribution of Na^+^/H^+^ exchanger regulatory factor 1 is determined by the PDZ-I domain and regulates the malignant progression of breast cancer

**DOI:** 10.18632/oncotarget.8751

**Published:** 2016-04-15

**Authors:** Guifang Du, Yanan Gu, Chengcheng Hao, Zhu Yuan, Junqi He, Wen G. Jiang, Shan Cheng

**Affiliations:** ^1^ Department of Biochemistry and Molecular Biology, Capital Medical University, Beijing 100069, China; ^2^ Beijing Key Laboratory of Cancer & Metastasis Research, Capital Medical University, Beijing 100069, China; ^3^ Department of General Surgery, Beijing Friendship Hospital, Capital Medical University, Beijing 100050, China; ^4^ Cardiff China Medical Research Collaborative, Cardiff University School of Medicine, Cardiff CF14 4XN, UK

**Keywords:** breast cancer, NHERF1, PDZ, cellular distribution, mutation

## Abstract

The oncogenic role of ectopic expression of Na^+^/H^+^ exchanger regulatory factor 1 (NHERF1) was recently suggested. Here, we show that NHERF1 was upregulated in high grades compared with low grades. Increased NHERF1 expression was correlated with poor prognosis and poor survival. NHERF1 expression was higher in the nucleus of cancer cells than in contiguous non- mammary epithelial cells. A novel mutation, namely *NHERF1* Y24S, was identified in human breast cancer tissues and shown to correspond to a conserved residue in the PDZ-I domain of NHERF1. Truncation and mutation of the PDZ-I domain of NHERF1 increased the nuclear distribution of the NHERF1 protein, and this redistribution was associated with the malignant phenotype of breast cancer cells, including growth, migration, and adhesion. The present results suggest a role for NHERF1 in the progression of breast cancer mediated by the nuclear distribution of the NHERF1 protein, as determined by the truncation or key site mutation of the PDZ-I domain.

## INTRODUCTION

Na^+^/H^+^ exchanger regulatory factor 1 (NHERF1, also known as EBP50) was first identified as an essential cofactor for cyclic AMP inhibition of Na^+^/H^+^ exchange in the rabbit renal brush border membrane [1]. It is a scaffold protein that is highly expressed in the apical membrane of polarized epithelial cells [2]. NHERF1 consists of two tandem PDZ domains followed by an ezrin-radixin-moesin (ERM)-binding region [3]. NHERF1 can bind to ion transporters, G protein-coupled receptors, and cytoskeleton-associated ERM proteins via these domains, and has been implicated in the regulation of diverse biological processes associated with ion transport and second messaging cascades, as well as in the maintenance of cell polarity [4].

Recent evidence indicates that NHERF1 binds many cancer-related proteins, such as phosphate and tensin homolog (PTEN) [5, 6], neurofibromatosis 2 [7], spleen tyrosine kinase [7], platelet-derived growth factor receptor (PDGFR) [8], epidermal growth factor receptor (EGFR), and β-catenin, suggesting its possible involvement in carcinogenesis [7, 9, 10]. NHERF1 mRNA levels vary among different human tissues, and NHERF1 is upregulated in several human s, including hepatocellular carcinoma (HCC) [11], breast cancer [12], and colorectal cancer [13]. Western blot and immunohistochemical analyses of a series of and contiguous non-involved breast tissues from the same patients showed that NHERF1 is highly overexpressed in tissues and associated with aggressive clinical characteristics and poor prognosis [14].

The heterogeneous and differential expression of NHERF1 is involved in the progression of several types [15, 16]. NHERF1 expression has been identified in tissues with polarized epithelia, with a main intracellular distribution at the apical luminal membranes of epithelial cells [17]. Alterations in the apical membrane localization of NHERF1 contribute to colorectal cancer through the disruption of epithelial morphology [18]. Ectopic cytoplasmic NHERF1 expression exacerbates the transformed phenotype by increasing cell proliferation [18]. Nuclear NHERF1 expression, which is present in the early stages of carcinogenesis in colorectal cancer and is correlated with poor prognosis, may contribute to the onset of the malignant phenotype [19, 20]. In a HCC model, NHERF1 was found to contribute to transcriptional regulation by interacting with and stabilizing the β-catenin protein. Stabilized β-catenin translocates and associates with different transcriptional factors, acting as a transactivator. NHERF1 and β-catenin colocalize in the nucleus of HCC cells, and NHERF1 is suggested to function as a positive regulator of Wnt signaling and contribute to the malignant phenotype [11, 21]. A heterogeneous distribution of NHERF1 expression was also observed in the normal breast, and in *in-situ* and invasives, metastatic lymph nodes and distant metastases [22]. Cytoplasmic NHERF1 expression progressively increases in cells from ductal carcinoma *in situ* (DCIS) to invasive and metastatic tissues, and the upregulation of cytoplasmic NHERF1 protein expression is accompanied by a progressive and significant decrease in membranous NHERF1 expression [22, 23]. These data indicate that NHERF1 may be useful as a marker of clinical relevance in cancer patients based on its expression and cellular distribution. However, the mechanism regulating the cellular distribution of the NHERF1 protein remains unclear.

In the current study, we investigated the expression pattern and cellular distribution of NHERF1 in human breast cancer tissues. The structural factors determining the cellular distribution of NHERF1 and the effects of its ectopic expression on breast cancer cells were also investigated to gain insight into the relationship between NHERF1 distribution and function, and to improve our understanding of the role of NHERF1 in development and progression.

## RESULTS

### NHERF1 expression was associated with the clinical status of breast cancer

**Figure 1:**
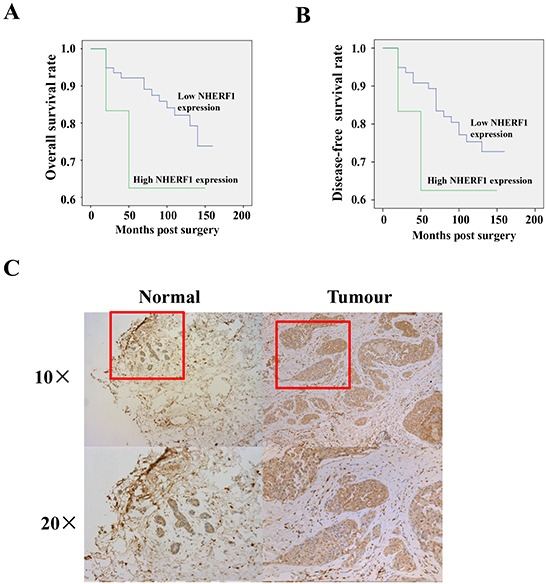
Expression of NHERF1 in normal and cancerous human breast tissues High expression of NHERF1 was associated with shorter overall **A.** and disease-free **B.** survival of patients with breast cancer. The NHERF1 protein was readily detected in both normal and cancerous epithelial cells at a similar level, but not in surrounding stromal cells by IHC **C.** *p<0.05, **p<0.01.

The correlation between NHERF1 expression and the clinical status of breast cancer patients is summarized in Table 1. NHERF1 transcript levels were increased in high grades compared with low grades (*p* = 0.0005, grade 3 *vs.* grade 1; *p* = 0.02, grade 3 *vs.* grade 2). NHERF1 upregulation was associated with poor prognosis (*p* = 0.04, NPI-3 *vs.* NPI-1; *p* = 0.002, NPI-3 *vs.* NPI-2) and decreased overall survival (OS). The mean OS was 102.0 [(55.2–148.8, 95% confidence interval (CI)] months in patients with high NHERF1 expression levels (cut-off by median) and 136.2 (126.6–145.9, 95% CI) months in patients with low NHERF1 expression levels (Figure 1A). Similar results were obtained for disease-free survival (DFS), with a mean DFS of 102.0 (55.2–148.8, 95% CI) months in patients with high NHERF1 expression levels (cut-off by median) and 130.7 (120.2–141.3, 95% CI) months in patients with low NHERF1 expression levels (Figure 1B). No significant differences were observed in the OS (*p* = 0.19) and DFS (*p* = 0.33) curve analyses between patients with high and low NHERF1 expression. These results suggest the prognosis relevance of NHERF1 expression in breast cancer. However, there was no association between NHERF1 expression in breast cancer tissues and other clinical variables including TNM staging and Survival status. NHERF1 expression levels did not differ significantly between adjacent normal and breast cancer tissues (Table 1). NHERF1 protein expression was detected at similar levels in normal and cancerous epithelial cells, but not in surrounding stromal cells (Figure 1C).

**Table 1:** Quantitative PCR analysis of NHERF1 expression in human breast tissues

Clinical data	Grouping	No.	Mean ± SEM	*p*-value
Tissue sample	Normal	31	425±422	
	Tumor	115	432±313	0.69
Tumor grade	1	20	11.2±9.07	
	2	39	7.18±4.45	0.02 *(2 vs. 1)
	3	54	906±663	0.0005* (3 vs. 1) 0.02* (3 vs. 2)
Nottingham Prognostic Index (NPI)	1 (<3.4)	58	64.9±50	
	2 (3.4–5.4)	38	7.79±6.55	
	3 (>5.4)	15	3024±2346	0.04* (3 vs. 1) 0.002* (3 vs. 2)
TNM staging	1	61	656±583	
	2	37	221±152	0.03*
	3	7	1.86±1.22	0.50
	4	4	1.33±1.33	0.57
Survival status	Disease free	81	458±437	
	Recurrence	7	12.6±8.05	0.30
	Metastasis	5	979±979	0.56
	Death	14	305±286	0.15
ER alpha status	(−)	69	516±4288	
	(+)	35	8.62±51	0.63
ER beta status	(−)	83	431±3922	
	(+)	24	167±818	0.10

### Subcellular distribution of NHERF1 in breast cancer

**Figure 2:**
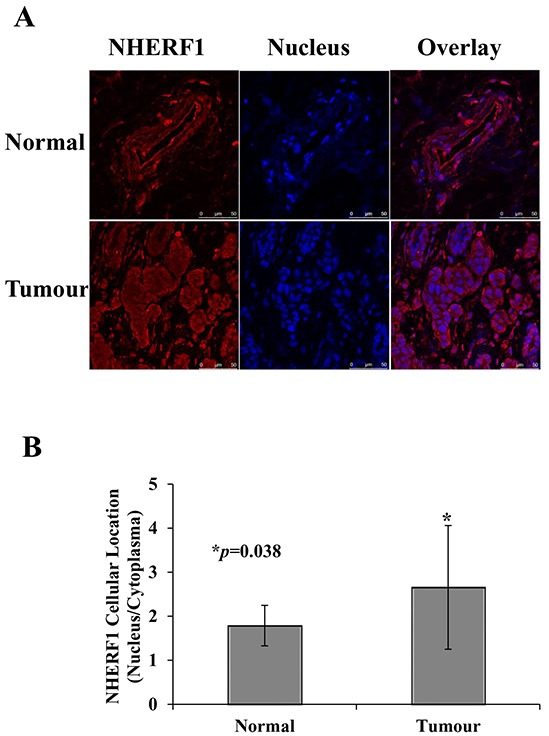
Subcellular distribution of NHERF1 in normal and cancerous human breast tissues Representative images of NHERF1 immunofluorescence staining are shown. In contiguous non-tumor breast tissues, NHERF1 showed mostly an apical membranous immunoreactivity in epithelial cells **A.** In the primary tumor and metastatic cells, NHERF1 mostly localized to the cytoplasm, with large areas of NHERF1 nuclear localization, especially where cells were not polarized (A). NHERF1 was upregulated in the nuclei of cancer cells, as shown by a higher nuclear/membranous ratio of NHERF1 staining in breast cancer cells than in normal mammary epithelial cells **B.** Scale bar, 50 μm; *p<0.05.

To examine the role of NHERF1 in breast cancer, NHERF1 expression was detected in the apical membrane, cytoplasm, and nucleus of tumor and non-tumor cells from patient tissues. Representative images of NHERF1 immunofluorescence staining are shown in Figure 2. NHERF1 immunoreactivity showed mostly an apical membranous and cytoplasmic distribution pattern in epithelial cells of adjacent non-tumor breast tissues (Figure 2A), whereas in cells, NHERF1 was detected in the cytoplasm, with large areas of NHERF1 nuclear localization, especially in cells that were not polarized (Figure 2A). NHERF1 protein expression was higher in the nucleus of cancer cells, as shown by a higher nucleus/cytoplasm ratio of NHERF1 staining in breast tumor cells when compared with that in non-tumor mammary epithelial cells (*p* = 0.038) (Figure 2B).

### The PDZ-I domain determined the distribution of NHERF1 in the membrane and cytoplasm

**Figure 3:**
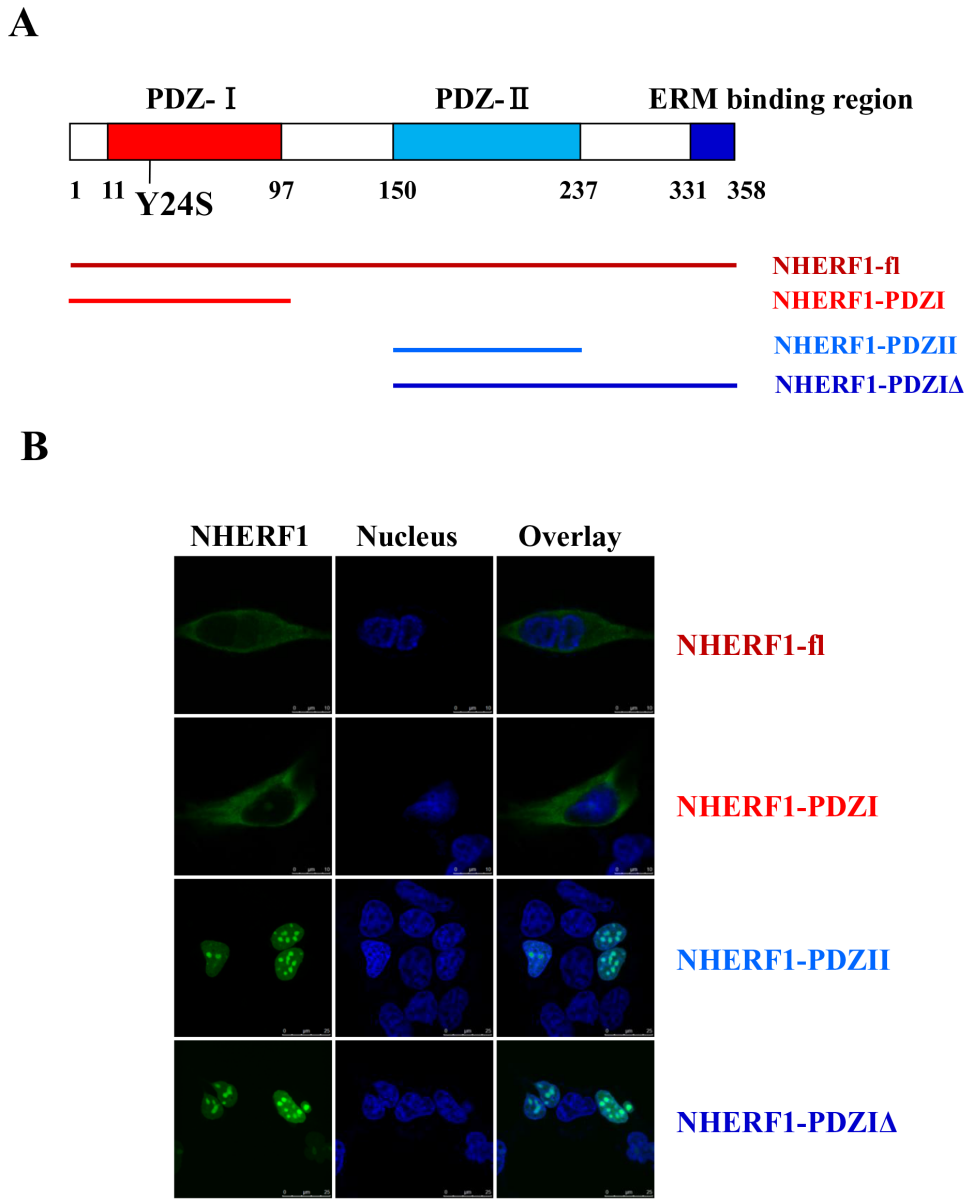
The PDZ-I domain mediated the distribution of NHERF1 in the membrane and cytoplasm NHERF1 and its truncated fragments **A.** are shown in green, the nucleus is shown in blue, and overlaid images are shown **B.** Scale bar, 50 μm. Wild-type NHERF1 and the PDZ-I domain of NHERF1 localize to the membrane and cytoplasm (B). Truncation of the PDZ-I domain resulted in a shift from cytoplasmic to nuclear localization of the NHERF1 protein (B).

To identify the structural determinant mediating the intracellular distribution of NHERF1, a set of NHERF1 truncated fragments were generated from the wild-type protein (Figure 3A). The imaging results showed that the wild-type NHERF1 and PDZ-I domain of NHERF1 were primarily located in the membrane and cytoplasm. Truncation of the PDZ-I domain resulted in a shift in the localization of a significant portion of the NHERF1 protein from the cytoplasm to the nucleus (Figure 3B). These data suggested that the PDZ-I domain determines the distribution of NHERF1 in the membrane and cytoplasm, which could contribute to the regulation of NHERF1 function in cells.

### The breast cancer-derived *NHERF1* Y24S mutation increased the nuclear expression of NHERF1

**Figure 4:**
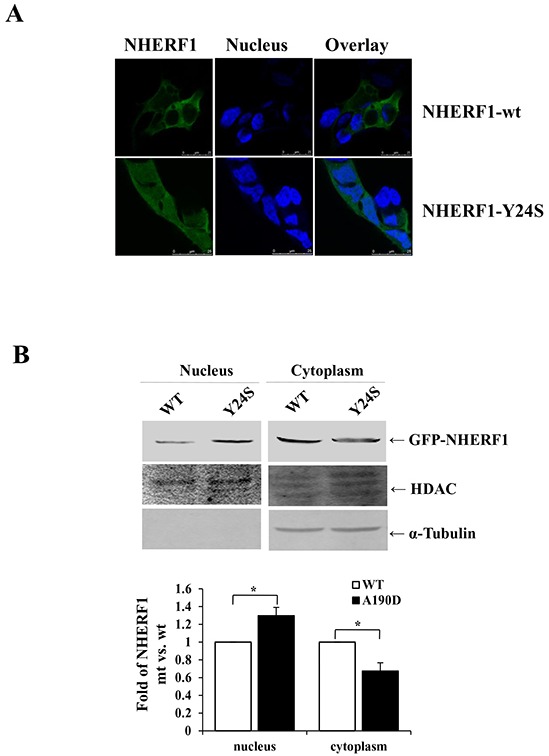
The cancer-derived *NHERF1* Y24S mutation increased the nuclear expression of NHERF1 GFP-NHERF1 wild-type and the Y24S mutant were expressed in HEK-293 cells. The NHERF1-Y24S mutation resulted in increased nuclear expression **A.** Scale bar, 50 μm. Western blot analysis confirmed that NHERF1-Y24S promoted the nuclear localization of NHERF1 **B.** The results represent the mean values ± SD of three independent experiments (B).*p<0.05.

The presence of mutation(s), particularly in the PDZ-I domain was investigated in 20 frozen breast cancer tissues. The coding region and the intron-exon junctions of the NHERF1 gene were analyzed by polymerase chain reaction-single strand conformation polymorphism (PCR-SSCP) (Supplementary Figure S1A) and confirmed by DNA sequencing (Supplementary Figure S1B). A previously unknown sequence variant (TAC to TCC) was identified in the first exon of *NHERF1* in a patient with medullary breast carcinoma, which would result in a switch of codon 24 (Tyr-Ser). The mutation corresponded to a conserved basic residue in the PDZ-I domain (Supplementary Figure S1C) [24]. Transient transfection of HEK-293 cells with GFP-NHERF1-Y24S resulted in an obvious shift in the localization of the NHERF1 protein from the cytoplasm to the nucleus, compared to that in cells transfected with GFP-NHERF1-wt (Figure 4A). Western blot analysis confirmed that NHERF1-Y24S increased the levels of nuclear NHERF1 (Figure 4B).

### Breast cancer-derived *NHERF1* Y24S mutation impaired the tumor-suppressor function of NHERF1

**Figure 5:**
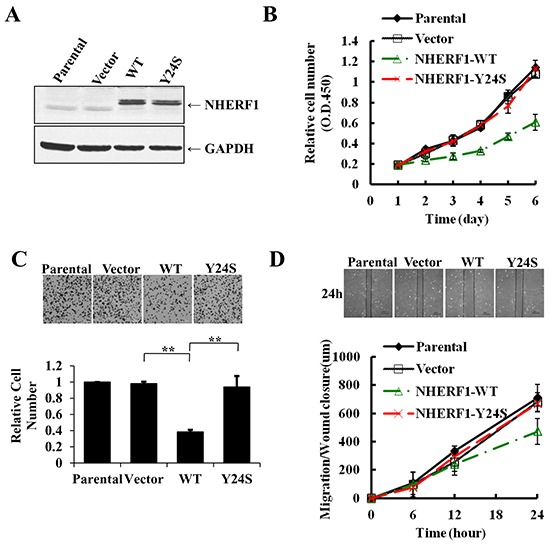
The breast cancer-derived *NHERF1* Y24S mutation abolished the antitumor effects of NHERF1 in MCF-7^ΔNHERF1^ cells Wild-type and Y24S mutant NHERF1 were stably transfected into MCF-7^ΔNHERF1^ cells and detected with an anti-NHERF antibody **A.** The Y24S mutation impaired the tumor-suppressor functions of NHERF1, including cell proliferation **B.** adhesion **C.** and migration **D.** The results represent the mean values ± SD of three independent experiments (B, C, D). *p<0.05, **p<0.01.

To further evaluate the biological role of the novel *NHERF1* mutation Y24S, wild-type and Y24S mutant *NHERF1* were stably transfected into MCF-7^ΔNHERF1^ (breast cancer cells in which NHERF1 was knocked down) cells and its expression was assessed by western blotting. The expression level of NHERF1 was considerably higher in stably transfected cells than in parental or vector control cells, and the Y24S mutation was expressed at similar levels than NHERF1-wt (Figure 5A). Evaluation of malignant phenotypes, including cell proliferation, adhesion, and migration, showed an approximately 50% lower cell proliferation rate in cells overexpressing NHERF1-wt than in parental or vector control cells on day 6. Cell adhesion was reduced by up to 60% at 40 min, and cell migration was reduced by up to 35% at 24 h in NHERF1-wt overexpressing compared to parental or vector control cells. However, the NHERF1-Y24S mutation lost the tumor-suppressor effects observed in NHERF1-wt. (Figure 5B–5D). These results indicated that NHERF1-wt acted as a tumor suppressor in MCF-7^ΔNHERF1^ cells, and the breast cancer-derived *NHERF1* mutation Y24S abolished its tumor-suppressor effects.

### The *NHERF1* Y24S mutation abolished the inhibitory effect of NHERF1 on FBS-induced AKT and ERK activation

**Figure 6:**
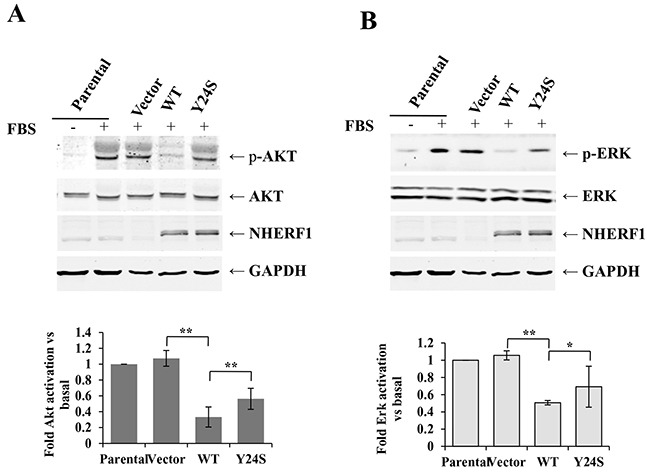
The Y24S *NHERF1* mutation resulted in the loss-of-function of the NHERF1-mediated inhibition of FBS-induced AKT and ERK activation MCF-7^ΔNHERF1^ cells stably transfected with various NHERF1 constructs were serum starved overnight, and then stimulated with or without FBS for 15 min. Immunoblotting was performed using anti-pS473AKT **A.** anti-AKT (A), anti-p-ERK **B.** and anti-ERK (B) antibodies. The signals were quantified by densitometry. Data are expressed as fold change with respect to stimulated parental cells. The results represent the mean values ± SD of three independent experiments (A, B). *p<0.05, **p<0.01.

Next, we detected the phosphorylation status of AKT and ERK in MCF-7^ΔNHERF1^ cells transfected with various NHERF1 constructs. Stimulation of MCF-7^ΔNHERF1^ parental or vector control cells with FBS for 15 min resulted in the marked phosphorylation of AKT (Figure 6A) and ERK (Figure 6B). NHERF1-wt overexpression significantly inhibited AKT and ERK activation, and this effect was partially abolished in cells expressing the *NHERF1-Y24S* mutation (Figure 6). No differences in AKT and ERK basal activation levels were detected among cells transfected with various NHERF1 constructs (data not shown). Taken together, these data suggested that the cancer-derived *NHERF1* mutation Y24S abolished the inhibitory effect of NHERF1 on FBS-induced AKT and ERK activation.

## DISCUSSION

In the present study, we found that NHERF1 was upregulated in high grade breast cancer patients and associated with poor prognosis (Table 1). Patients with high NHERF1 expression had a shorter survival time than those with low NHERF1 expression (Figure 1). These results, which were consistent with those of previous studies assessing NHERF1 expression in breast cancer [14] confirmed the oncogenic role of NHERF1 in breast cancer. However, there was no significant association between NHERF1 expression in breast cancer tissues and other clinic variables, which could be attributed to the large variation in the data. These results suggest that the effect of NHERF1 on the progression of breast cancer involves other factors in addition to its expression level. In humans, NHERF1 is expressed in many epithelial tissues and localizes mainly to the apical luminal membranes of epithelial cells [23]. NHERF1 alterations are correlated with the progression and invasiveness of human tumors, and its heterogeneous distribution is a common oncogenic event in carcinomas [10]. Although studies have investigated the correlation between cytoplasmic overexpression of NHERF1 and oncogenic progression, little is known about the involvement of nuclear NHERF1 in breast cancer. In the present study, we examined the effects of ectopic nuclear expression of NHERF1 in human breast cancer tissues. Nuclear NHERF1 was upregulated in tumor cells, as shown by a higher nuclear/cytopasmic ratio of NHERF1 immunofluorescence staining in breast tumor cells than in adjacent non-tumor mammary epithelial cells (*p* = 0.038) (Figure 2). These results indicated that NHERF1 expression and cellular distribution may be useful as markers of clinical relevance in cancer patients. However, the mechanism regulating NHERF1 cellular distribution remains unclear, and little is known about the role of NHERF1 in the nucleus.

NHERF1 is a 358-residue protein comprised of two tandem PDZ domains and a C-terminal ERM bind region (Figure 3A). Experiments with truncated or mutated forms of NHERF1 in epithelial OKP cells and *in vivo* experiments in ezrin(−/−) mice support that NHERF1 is stabilized at the epithelial apical membrane through its interaction with ERM proteins [25]. Recent reports indicate an alternative role for PDZ-domain interactions in the recruitment of NHERF1 to the membrane. NHERF1 PDZ-I domain is involved in membrane recruitment through a phosphorylation switch of Ser77 [26]. In the present study, the PDZ-I domain was shown to play an important role in the membrane and cytoplasmic localization of NHERF1, as shown by the nuclear distribution of PDZ-I truncated NHERF1 proteins (NHERF1-PDZII and NHERF1-PDZIΔ) (Figure 3B). PDZ domains, which are common protein modules involved in protein–protein interactions, bind directly to the carboxyl (C)-terminal PDZ motifs of their ligands [27]. Certain NHERF1-associated proteins bind to the first PDZ (PDZ-I) domain, including membrane receptors, such as EGFR, PDGFR, β2AR, and CFTR, and cytoplasmic proteins such as PTEN and PLCβ [28–30]. The interaction of NHERF1 with these proteins could regulate the distribution of NHERF1 in the membrane and cytoplasm. In addition, the subcellular distribution of NHERF1 could affect the interaction between NHERF1 and downstream signaling proteins, which could impact the oncogenic role of NHERF1 in breast cancer.

The Tyr24 residue plays a key role in the formation of the three-dimensional pocket of the PDZ-I domain [31–34]. It directly interacts with the Leu 0 of the carboxylate group in the interaction with the C-terminal end of the binding proteins [32]. In the present study, a novel *NHERF1* sequence variant (TAC to TCC) resulting in a switch of codon 24 (Tyr-Ser) was identified in human breast cancer tissues (Suppl Figure 1). Mutation of codon 24 of *NHERF1* could change the three-dimensional structure of PDZ-I and impair protein interactions. Confocal microscopy showed that transient transfection of HEK-293 cells with GFP-NHERF1-Y24S resulted in a significant switch in the localization of the NHERF1 protein from the cytoplasm to the nucleus, compared with that in cells transfected with GFP-NHERF1-wt (Figure 4). Y24S mutation-induced alterations in the interaction between NHERF1 and its ligand proteins in the membrane and cytoplasm could decrease the recruitment of NHERF1 to the membrane and cytoplasm, resulting in its relocalization to the nucleus.

Clinical studies show an association between NHERF1 overexpression and the malignant progression of cancer [12]; however, contradictory results were reported in many *in vitro* studies indicating a tumor suppressor role of NHERF1. NHERF1 overexpression plays a tumor suppressor role in breast cancer cell lines, as shown by the inhibitory effect of NHERF1 on canonical Wnt signaling and Wnt-dependent cell proliferation in MCF-7 and MDA MB-231 cells [35]; NHERF1 knockdown accelerates cell cycle progression in parallel with increased expression of cyclin E and elevated Rb phosphorylation levels [36]. Our previous studies also showed that overexpression of NHERF1 reduces cell proliferation, motility, and invasion of low-NHERF1-expressing SKMES-1 cells, and knockdown of NHERF1 enhances the migratory and invasive ability of MCF-7 cells [37]. These contradictory results may be due to differences in the expression patterns of NHERF1 in *in vivo* and *in vitro* models; for example, the subcellular expression pattern may be different in nature and in artificial cells, resulting in the activation of different signaling pathways and the expression of different cell phenotypes. In the present study, *in vitro* functional experiments showed that overexpression of NHERF1 reduced cell proliferation, motility, and adhesion in low-NHERF1-expressing MCF-7^ΔNHERF1^ cells (Figure 5).

The identification of a novel mutation in this study, namely the *NHERF1* Y24S mutation, supports the role of the NHERF1 protein and the importance of its cellular distribution. Truncation (Figure 3) and mutation (Figure 4) of the PDZ-I domain promoted the nuclear localization of the NHERF1 protein, suggesting that NHERF1 functions through the PDZ-II domain in the nucleus. Although only a few proteins specifically interact with the second PDZ (PDZ-II) domain of NHERF1, many nuclear transcription factors are involved, such as β-catenin [11,21] and Yap 65 [38]. The interaction of NHERF1 with stabilized β-catenin functions in transcriptional regulation, suggesting its role as a transactivator [21]. The function of NHERF1 in the nucleus remains unclear; however, the nuclear localization of NHERF1 could promote the interaction between NHERF1 and nuclear transcription factors to regulate the expression of related signaling proteins, resulting in the transformation of cell phenotypes.

Overall, it was showed that NHERF1 upregulation was associated with poor prognosis and decreased survival time of patients with breast cancer. Nuclear NHERF1 protein expression was higher in cancer cells than in adjacent non-tumor mammary epithelial cells. The truncation and mutation of the PDZ-I domain increased the nuclear distribution of NHERF1. NHERF1-wt overexpression reversed the malignant phenotypes of MCF-7^ΔNHERF1^ cells, including cell proliferation, migration, and adhesion. The breast cancer-derived NHERF1 mutation Y24S inactivated the inhibitory effect of NHERF1 on FBS-induced AKT and ERK activation, and resulted in the partial loss of its tumor-suppressor effects. These results support the role of NHERF1 in tumor development and progression, which could be promoted by the nuclear expression of NHERF1. The nuclear expression of NHERF1 could be determined by the truncation or key site mutation of the PDZ-I domain.

## MATERIALS AND METHODS

### Cell lines and culture

A human breast cancer cell line with low NHERF1 expression (MCF-7^ΔNHERF1^) was generated previously [37] by stably expressing a ribozyme targeted to NHERF1. HEK-293 cells were obtained from American Type Culture Collection (ATCC, Manassas, VA, USA). All cells were maintained in Dulbecco's Modified Eagle's Medium (DMEM) supplemented with 10% fetal calf serum, penicillin, and streptomycin (Gibco BRC, Paisley, Scotland) in an incubator at 37°C, 5% CO_2_, and 95% humidity.

### Human breast specimens

A total of 146 breast samples were obtained from breast cancer patients (31 were adjacent normal breast tissues (> 2 cm to the tumor) and 115 were breast cancer tissues). These tissues were collected immediately after mastectomy, and snap-frozen in liquid nitrogen, with the approval of the Local Ethical Committee. Background normal mammary tissues were removed from the same patients. The pathologist verified normal background and cancer specimens, and background samples were confirmed to be free from tumor deposits. The median follow-up for the cohort was 120 months (June 2004). The relevant information is provided in Table 1. Survival time was calculated from the date of surgery, and recurrence or metastasis was counted on the date of diagnosis thereof.

### RNA preparation and real-time quantitative polymerase chain reaction (QPCR)

Total cellular RNA was isolated from the homogenized breast samples using the ABgene Total RNA Isolation Reagent and following the protocol provided (Advanced Biotechnologies Ltd., Epsom, Surrey, UK). cDNA was generated from 1 ug of each RNA sample and reverse transcribed using a transcription kit (Sigma, St. Louis, MO, USA). Quantitative analysis of NHERF1 mRNA expression in breast tissues was determined by QPCR using Amplifor™-based technologies, in which a 6-carboxy-fluorescine-tagged Uniprimer™ (Biosearch Technologies, Inc., Petaluma, CA, USA) was used as a probe together with a pair of target-specific primers and reverse primer with an additional Z-sequence (actgaacctgaccgtaca) (*NHERF1* QPCR primers – sense: AGGGAAACTGACGAGTTCTT; antisense: ACTGAACCTGACCGTACATTCACGACTGTTCTCCTTCT). Real-time QPCR conditions were 95°C for 15 min, followed by 60 cycles at 95°C for 20 s, 55°C for 30 s and 72°C for 20 s. The quality of cDNA samples was verified using β-actin as a housekeeping gene (*β-actin* QPCR primers – sense: CATTAAGGAGAAGCTGTGCT; antisense: ACTGAACCTGACCGTACAGCTCGTAGCTCTTCTCCAG). The epithelial content within the tumors was taken into account by normalizing *NHERF1* levels against cytokeratin 19 (*CK19* primer details- sense: CAGGTCCGAGGTTACTGAC; antisense: ACTGAACCTGACCGTACACACTTTCTGCCAGTGTGTCTTC).

### Immunohistochemical and immunofluorescence staining of breast specimens

Frozen sections of breast tumors (n=25) (8 Grade-1, 8 Grade-2, and 9 Grade-3) and background tissues (n=25) were selected from the tissue bank and cut at a thickness of 6 μm using a cryostat. The sections were fixed in a mixture of 50% acetone and 50% methanol and then placed in ‘Optimax’ wash buffer for 5-10 min to rehydrate. Sections were incubated for 20 min in a horse serum blocking solution and probed with monoclonal mouse anti-human NHERF1 primary antibody (1:200) (Santa-Cruz Biotechnologies, Santa Cruz, CA, USA). This study employed controls that omitted the primary and secondary antibodies. For immunohistochemical staining, following extensive washings, sections were incubated for 30 min in the secondary biotinylated antibody (1:100) (Multilink Swine anti-goat/mouse/rabbit immunoglobulin, Dako Inc., Copenhagen, DK). Following washings, the Avidin Biotin Complex (Vector Laboratories, Peterborough, UK) was then applied to the sections, followed by extensive washing steps. Diamino benzidine chromogen (Vector Labs) was then added to the sections, and incubated in the dark for 5 min and counterstained by hematoxylin. Sections were then dehydrated in ascending grades of methanol before clearing in xylene and mounting under a cover slip.

For immunofluorescence staining, the TRITC-conjugated secondary antibody (Invitrogen, Paisley, Scotland, UK) was subsequently added to the slides, and the slides were incubated on a shaker platform in the dark for 1 h. After washing three times to remove the unbound secondary antibody, cell nuclei were stained with Hoechst 33258 (Sigma). The slides were finally mounted with FluorSave™ (Calbiochem-Novabiochem Ltd., Nottingham, UK) and visualized with a confocal microscope (Leica Microsystems LAS AF-TCS SP5. Wetzlar, Germany). Immunostaining reaction intensity and area [integral optical density (IOD)] for NHERF1 were separately assessed by 2 independent authors who were blinded to the tissue grouping using computer-assisted morphological analysis. Image Pro Plus 6.0 (Media Cybernetics, Inc., Rockville, MD, USA) was used to calculate the intensity and extent of staining for NHERF1, and the ratio of the nucleus staining area to the cytoplasma area of the image in the different tissues. A total of 10 microscopic fields were randomly selected, and their images were cropped. Results were expressed as the mean ± standard deviation (SD).

### Mutational analyses

The genomic DNA from 20 frozen breast cancer tissues was extracted using the DNeasy kit (Qiagen, Hilden, Germany). All six exons of the *NHERF1* gene were amplified by PCR using primers corresponding to the neighboring intronic sequences, and further analyzed by SSCP as previously described [39]. Primers were: exon 1 (forward, 5ʹ-tgggacacctgcttgcttg-3ʹ; reverse, 5ʹ-atcctcctcccactccatg-3ʹ); exon 2 (forward, 5ʹ-aattgctgtgtagggatctag-3ʹ; reverse, 5ʹ-ggaagagagcgagaagcatc-3ʹ); exon 3 (forward, 5ʹ-actgcaaa ctggctgagaac-3ʹ; reverse, 5ʹ-tggctcacatccctgacttg-3ʹ); exon 4 (forward, 5ʹ-attcatggtgggtggtagtc-3ʹ; reverse, 5ʹ-caccttctg atctgtctcatg-3ʹ); exon 5 (forward, 5ʹ-aggctcaggaggtgggaac-3ʹ; reverse, 5ʹ-ggcttcctgtaacccagttg-3ʹ); and exon 6 (forward, 5ʹ-agccgcattctgttcttgtg-3ʹ; reverse, 5ʹ-gaaaaaggtggggtgg aatg-3ʹ). The candidates of mutant gene were then identified by DNA sequencing.

### Preparation of plasmids and fusion proteins

*NHERF1* cDNA was cloned into pEASY™-M2 and pcDNA3.1 vector by using pEASY-Blunt M2 Expression Kit (TransGen, Beijing, China) and pcDNA3.1/CT-GFP TOPO TA expression kit (Invitrogen) to obtain the NHERF1 and GFP-NHERF1 expression constructs, according to the manufacturer's instructions. The constructs of the NO-tagged and GFP-tagged *NHERF1-Y24S* mutant were generated from the wild type with the use of the Fast MultiSite Mutagenesis System kit (TransGen). The PDZ-I domain (amino acids 1–97 of the human NHERF1 protein, NHERF1-PDZI), the PDZ-II domain (amino acids 150–237 of human NHERF1 protein, NHERF1-PDZII) and the NHERF PDZ-I domain truncation fragment (amino acids 150–358 of the human NHERF1 protein, NHERF1-PDZIΔ) were amplified by PCR and subcloned into pcDNA3.1/CT-GFP as GFP fusion proteins.

### Cytoplasmic and nuclear extracts

Cells were cultured in 10-cm plates for 48 h. After washing with cold PBS once, cells were harvested in 1000 μl of cold PBS. Cells were centrifuged (2 min, 500 g) at 4°C, washed twice with cold PBS, and resuspended in 400 μl of cold lysis buffer (10 mM HEPES, 50 mM NaCl, 5 mM EDTA, 1 mM Benzamidine, 0.5% Triton X-100). The lysates were then solubilized via vortex for 15 s and placed on ice for 20 min. The resulting homogenate was centrifuged for 1 min (2,000 g) at 4°C and the supernatant was kept as the cytoplasmic fraction. The precipitate was resuspended in 40 μl nuclear lysis buffer. The lysates were placed on ice for 40 min and solubilized via vortex for 15 s every 10 min. The resulting homogenate was centrifuged for 10 min (16,000 g) at 4°C and the supernatant was kept as the nuclear fraction. Samples were stored at −80°C.

### Western blotting and antibodies

Western blotting was performed as described previously [40]. The anti-NHERF1and anti-pS473AKT antibodies were purchased from Cell Signaling Technology (Danvers, MA, USA); anti-AKT antibody was from Sigma; anti-α-Tubulin, anti-HDAC and anti-GAPDH were from Santa Cruz Biotechnology; anti-GFP and anti-His antibodies were from MBL (Tokyo, Japan).

### Cell proliferation assay

Cell viability was evaluated using a nonradioactive cell counting kit (CCK-8, Dojindo, Kamimashiki-gun, Kumamoto, Japan) according to the manufacturer's instructions. Cells were seeded in a 96-well plate at a density of 3000 cells per well, and after every 24 h, the CCK-8 reagent was added to each well and the plates were incubated for an additional 1 h at 37°C. Cell viability was measured as the absorbance at 450 nm with an Elx800™ spectrophotometer (BioTek, Winooski, VT, USA).

### Wound-healing assay

The migratory properties of cells were assessed by wound-healing assay. Cells were seeded at a density of 2 × 10^5^ cells/well into a 24-well plate and allowed to reach confluence. The layer of cells was then scraped with a fine gauge needle to create a wound of approximately 1500 μm. Images of the wound were recorded under a phase contrast microscope at different times (0, 6, 12, and 24 h). Wound closure/cell migration was evaluated with motion analysis and line morphometry software (Optimus 6).

### Cell adhesion assay

A 96-well plate was pre-coated with 5 μg/well of Matrigel (BD Biosciences, Oxford, UK). Cells were seeded at a density of 2 × 10^5^ cells/well. After incubation at 37°C with 5% CO_2_ for 40 min, the cells were fixed, stained, and quantified as described previously [41].

### Statistical analysis

SPSS version 16.0 (SPSS, Inc., Chicago, IL, USA) was used for statistical analyses. The results were assessed using non-paired (two-sided) Student's *t*-test and Mann-Whitney U-test. Overall and disease-free survival rates were calculated using the Kaplan-Meier method. The log-rank test was utilized to compare the survival rates between groups with varying NHERF1 expression levels. The association of expression with the clinical and pathological features was analyzed using one-way ANOVA. A *p*-value <0.05 was defined as statistically significant.

## SUPPLEMENTARY FIGURE


