# Supplementary material for: The cellular distribution of Na+/H+ exchanger regulatory factor 1 is determined by the PDZ-I domain and regulates the malignant progression of breast cancer
Source: Oncotarget. 2016 Apr 15;7(20):29440–53. doi: 10.18632/oncotarget.8751 (PMC5045408; doi:10.18632/oncotarget.8751)
Supplement: Supplementary file 1 [file oncotarget-07-29440-s001.pdf]

SUPPLEMENTARY FIGURE

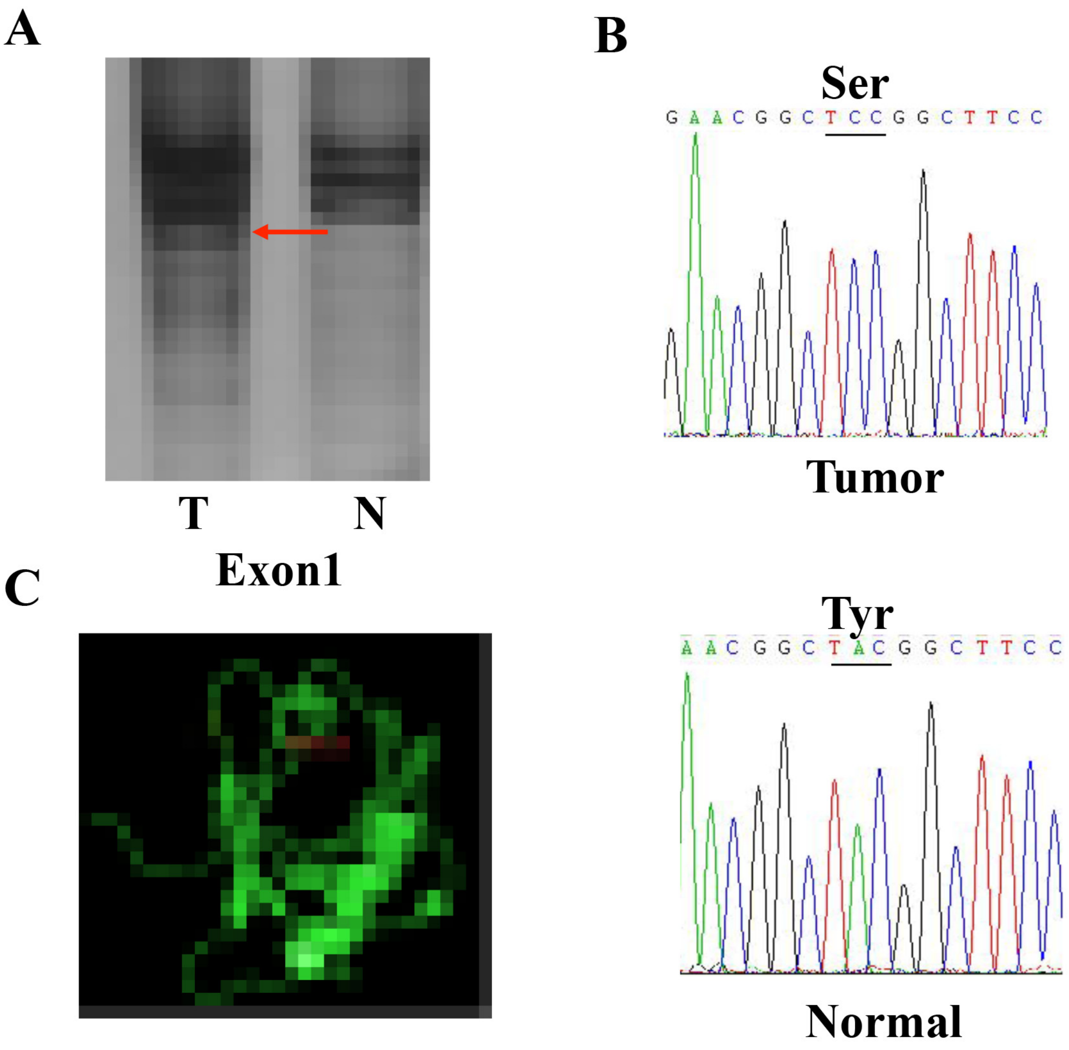

Supplementary Figure S1: A novel *NHERF1* mutation in patients with medullary breast carcinoma. A *NHERF1* mutation in exon 1 was detected by SSCP analysis. Abnormal migrational SSCP band patterns in the mutant are shown as compared with those in controls **A.** A previously unreported sequence variant (TAC to TCC) was identified by sequencing, which would result in a switch of codon 24 (Tyr to Ser) **B.** The mutation corresponded to a conserved basic residue in the PDZ I domain of NHERF1. The figure was generated with the software Pymol **C.**
